# Role of Immediate-Early Genes in Synaptic Plasticity and Neuronal Ensembles Underlying the Memory Trace

**DOI:** 10.3389/fnmol.2015.00078

**Published:** 2016-01-05

**Authors:** Keiichiro Minatohara, Mika Akiyoshi, Hiroyuki Okuno

**Affiliations:** Medical Innovation Center/SK Project, Graduate School of Medicine, Kyoto UniversityKyoto, Japan

**Keywords:** immediate-early gene, c-*fos*, *Arc*, synaptic plasticity, neuronal ensemble, memory trace

## Abstract

In the brain, neuronal gene expression is dynamically changed in response to neuronal activity. In particular, the expression of immediate-early genes (IEGs) such as *egr*-1, c-*fos*, and *Arc* is rapidly and selectively upregulated in subsets of neurons in specific brain regions associated with learning and memory formation. IEG expression has therefore been widely used as a molecular marker for neuronal populations that undergo plastic changes underlying formation of long-term memory. In recent years, optogenetic and pharmacogenetic studies of neurons expressing c-*fos* or *Arc* have revealed that, during learning, IEG-positive neurons encode and store information that is required for memory recall, suggesting that they may be involved in formation of the memory trace. However, despite accumulating evidence for the role of IEGs in synaptic plasticity, the molecular and cellular mechanisms associated with this process remain unclear. In this review, we first summarize recent literature concerning the role of IEG-expressing neuronal ensembles in organizing the memory trace. We then focus on the physiological significance of IEGs, especially *Arc*, in synaptic plasticity, and describe our hypotheses about the importance of *Arc* expression in various types of input-specific circuit reorganization. Finally, we offer perspectives on *Arc* function that would unveil the role of IEG-expressing neurons in the formation of memory traces in the hippocampus and other brain areas.

## Introduction

Optogenetics and pharmacogenetics have become indispensable techniques to interrogate neuronal populations and circuits that underlie specific physiological functions and behavior (Deisseroth, 2015; Urban and Roth, 2015). In particular, combination of these techniques with cellular labeling/tagging specific to active neuronal ensembles has allowed elucidation of the physiological significance of neuronal ensembles in memory formation, storage, and recall. This review aims to provide an overview of recent understanding of memory traces in the brain and to help understand the molecular and cellular mechanisms underlying the memory trace by discussing two major topics: (I) immediate-early gene (IEG)-expressing neuronal ensembles as memory traces, and (II) the roles of *Arc* in synaptic plasticity and memory formation. Below, we begin with a brief background describing relationships between memory, synaptic plasticity, and IEGs.

Long-lasting forms of synaptic plasticity such as long-term potentiation (LTP) and long-term depression (LTD) are fundamental cellular mechanisms underlying learning and memory (Bliss and Collingridge, 2013). Induction of LTP occurs concomitantly with learning in the hippocampus of freely moving animals and is known to preclude subsequent electrical induction of LTP in the hippocampus (Whitlock et al., 2006). Conversely, prior massive induction of hippocampal LTP is also known to interfere with spatial memory formation (Barnes et al., 1994). A recent study has demonstrated that *in vivo* artificial induction of LTD impaired recall of associative memory, which was restored by subsequent LTP induction (Nabavi et al., 2014). Taken together, these findings suggest that a causal relationship exists between long-term synaptic plasticity and memory processes.

The molecular mechanisms underlying LTP have also been extensively investigated. Following plasticity-inducing synaptic input, Ca^2+^ entry through *N*-methyl-D-aspartate (NMDA)-type receptors (NMDARs) plays a critical role in the onset of LTP via facilitation of α-amino-3-hydroxy-5-methyl-4-isoxazolepropionic (AMPA) receptor (AMPAR) recruitment to the potentiated post-synaptic sites (Collingridge et al., 1983; Kessels and Malinow, 2009). Furthermore, NMDAR-associated Ca^2+^ influx influences stabilization of LTP through activation of intracellular signaling cascades that subsequently promote mRNA and protein synthesis (Kandel et al., 2014). The blockade of these pathways using NMDAR antagonists (e.g., APV) or protein synthesis inhibitors (e.g., anisomycin) results in failure of the establishment of persistent LTP and impairment in formation of long-term memory (LTM; Gold, 2008; Redondo and Morris, 2011). Although these studies suggest that specific genes, induced during LTP, encode plasticity-related proteins (PRPs) required for LTP maintenance and memory formation, the identity of these genes remains unknown. A subset of plasticity-evoked, stimuli-induced genes, known as IEGs, has been implicated in the above events because of their rapid and transient responsiveness to synaptic activation (Okuno, 2011). For example, expression of IEGs such as *egr*-1 (*zif*268/*krox*-24), c-*fos*, and *Arc* (*arg*3.1), is rapidly upregulated after neuronal activation associated with pharmacologically induced convulsive and sensory stimuli (Morgan et al., 1987; Saffen et al., 1988; Link et al., 1995; Lyford et al., 1995). Behavioral tasks also induce IEG expression in neurons; such IEG-expressing neurons are distributed across a wide variety of brain regions (Rosen et al., 1998; Guzowski et al., 1999; Vann et al., 2000; Hall et al., 2001; Ramirez-Amaya et al., 2005). In the following sections, we describe studies analyzing the behavioral-induced IEG expression related to memory trace formation in more detail.

## IEG-Expressing Neuronal Ensembles as Memory Traces

### Induction of IEG Expression in Cell Ensembles Related to Cognitive Information Processing

Immediate-early genes such as *Arc*, c-*fos*, and *egr*-1 are induced in specific brain regions during neuronal activity associated with behavioral tasks. In the hippocampus, a center of declarative memory formation, rapid transcription of IEGs occurs during hippocampal-dependent learning paradigms including Morris water maze, novel environment exposure, and contextual fear conditioning (CFC; Guzowski et al., 1999, 2001; Vann et al., 2000; Hall et al., 2001; Ramirez-Amaya et al., 2005; Mamiya et al., 2009). *Arc* transcription is activated in a constant population (about 40%) of CA1 neurons following exposure to a novel environment (Guzowski et al., 1999, 2006; Vazdarjanova et al., 2002). This proportion is similar to the percentage of activated neurons mapped using electrophysiology (Guzowski et al., 2006), suggestive of a strong correlation between neuronal activity and *Arc* expression. In addition to the hippocampus, other brain regions also contain IEG-positive neurons activated during learning and memory. Fear conditioning results in rapid IEG expression in the lateral amygdala (Rosen et al., 1998; Hall et al., 2001; Reijmers et al., 2007; Ploski et al., 2008), suggesting that these IEG-expressing neurons may be associated with emotional memory formation (Ploski et al., 2008; Maddox and Schafe, 2011).

The RNA transcripts of several IEGs, including *Arc*, *egr*-1 and *homer*1a, first appear in the nucleus minutes after neuronal activation and are subsequently transferred to the cytoplasm (Guzowski et al., 1999). This temporally distinctive localization of IEG mRNA permits the differential labeling of activated neurons at different time points. This labeling method, known as cellular compartment analysis of temporal activity by fluorescent *in situ* hybridization (catFISH), has shown that sequential exposure to different environments induces IEG mRNA expression in distinct neuronal ensembles within the hippocampus, while sequential exposure to identical environments induces IEG mRNA in the same ensembles, indicating that activity-dependent IEG expression reflects spatial information processing in the hippocampus (Guzowski et al., 1999).

### Neuronal Ensembles with IEG Expression are Part of the Memory Trace

**FIGURE 1:**
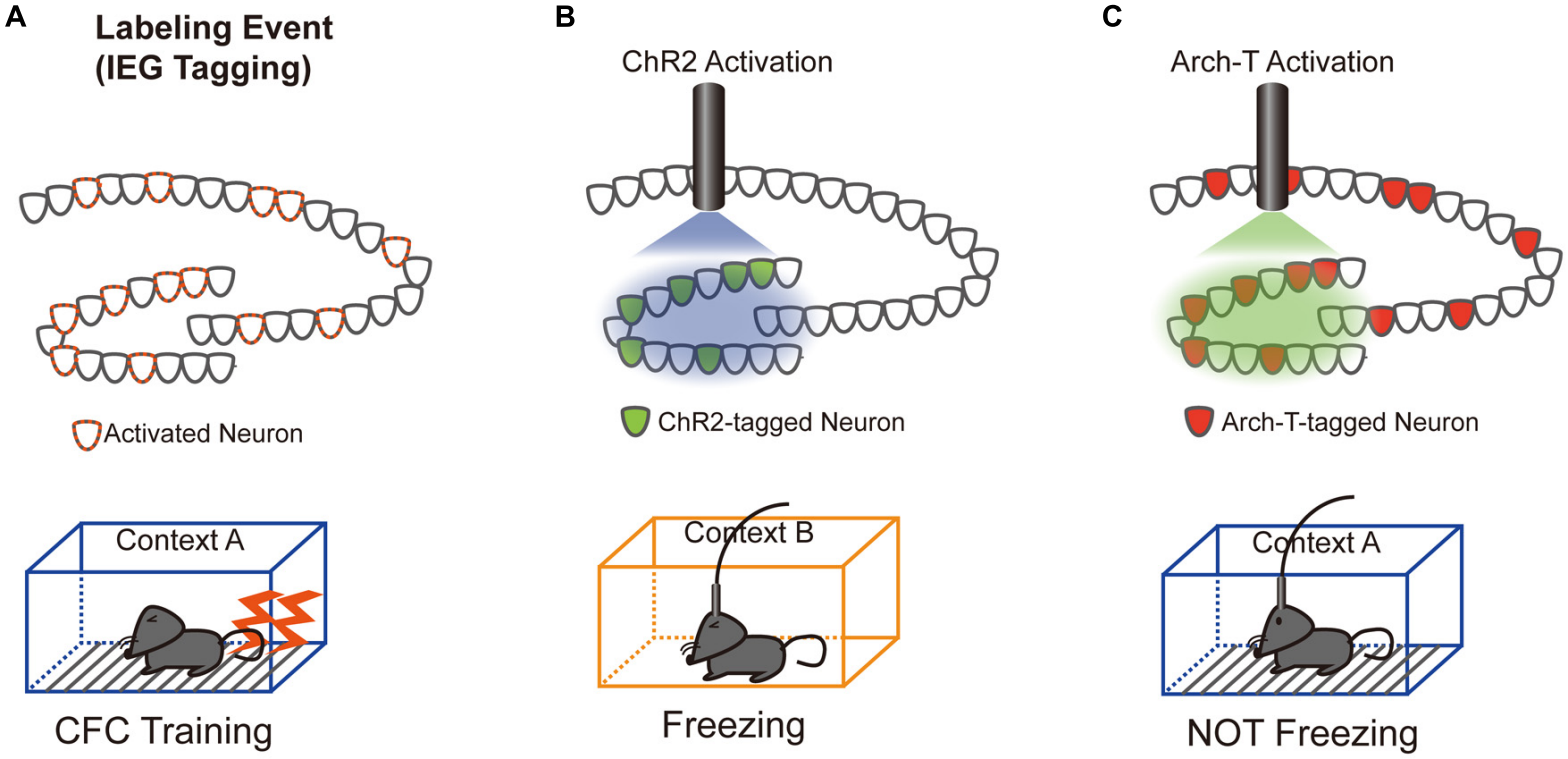
**Schematics of the optogenetic intervention experiments.** **(A)** DG neurons activated during contextual fear conditioning (CFC) training in a conditioned context (context A) were labeled with either Channelrhodopsin 2 (ChR2; **B**) or Archaerhodopsin (Arch-T; **C**), whose expression is controlled by IEG promoters. **(B)** Trained mice show freezing responses during unconditioned context (context B) exposure only when the c-*fos*-positive ensemble expressing ChR2 was reactivated by blue light illumination (Liu et al., 2012). **(C)** Consistent with the ChR2 reactivation data, trained mice show no freezing responses to conditioned context A only when neuronal activity of the *Arc*-positive ensemble expressing Arch-T was inhibited by green light illumination (Denny et al., 2014).

**FIGURE 2:**
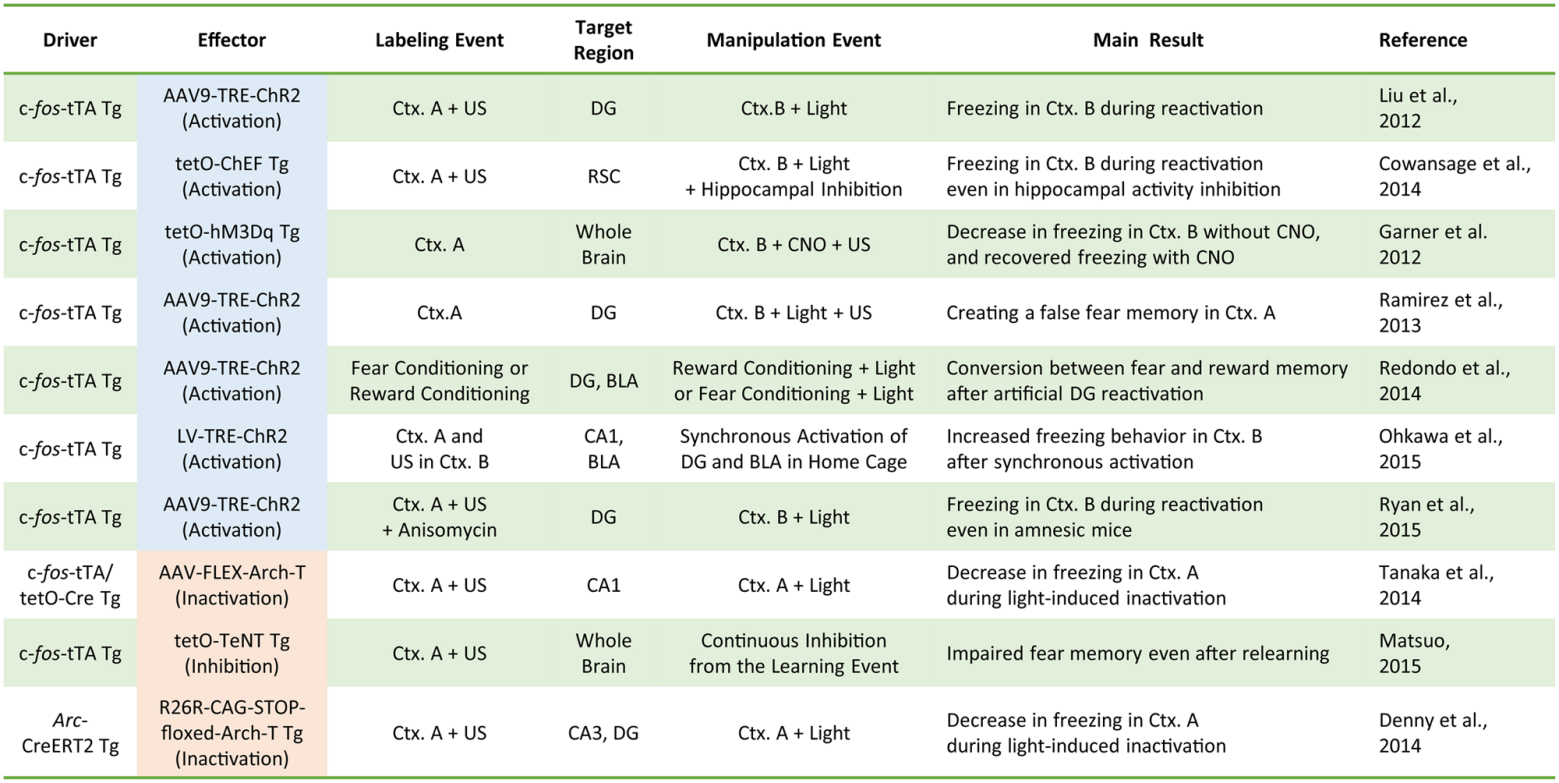
**A compendium of the current literature investigating the role of IEG-expressing neurons in memory formation.** Driver transgenes promote the expression of the effector proteins that can manipulate neuronal activity. Labeling events induce effector protein expression via the driver transgenes. During activity manipulation events, IEG-expressing neurons are activated or inactivated by applying the trigger (e.g., light or clozapine-*N*-oxide; CNO). Tg, transgenic animal; AAV, adeno-associated virus; LV, lentivirus; tTA, tetracycline transactivator; tetO, tetracycline operator; TRE, tetracycline response element; ChEF, channelrhodopsin chimeric variant; Ctx, context; US, unconditioned stimulus (e.g., electrical foot shock); FLEX, flip-excision (double floxed system); TeNT, tetanus toxin.

Our understanding of the role of IEG-expressing neuronal ensembles in fear memory formation has been dramatically enhanced by recent studies using optogenetic and pharmacogenetic manipulation of neuronal activity in these ensembles. The CFC paradigm is designed to create an association between a neutral conditioned stimulus (e.g., chamber exposure) and an aversive unconditioned stimulus (e.g., foot shock; LeDoux, 1992). If an animal forms a fear memory through conditioning, freezing behavior is observed when the animal is re-exposed to the conditioned stimulus alone. Activated neurons during CFC transiently express IEGs (Hall et al., 2001; Mamiya et al., 2009). Moreover, expression of light-gated ion channels such as Channelrhodopsin 2 (ChR2) and Archaerhodopsin (Arch-T) or ligand-gated G-protein-coupled receptors such as designer receptors exclusively activated by designer drugs (DREADDs) in neurons under the control of IEG promoters permits manipulation of the activity of IEG-expressing neurons responding to specific training experiences (**Figure 1**; Neves et al., 2008). **Figure 2** summarizes recent evidence concerning optogenetic and pharmacogenetic manipulation of IEG-expressing neurons. The seminal study by Liu et al. (2012) that demonstrated involvement of IEG-expressing neurons in the memory trace used two transgene components, c-*fos*-tTA transgenic (tg) mice and TRE-ChR2 adeno-associated viral (AAV) vectors, by which ChR2 was expressed via a c-*fos* promoter only during an off-doxycycline (off-Dox) phase (Liu et al., 2012). These mice were subjected to CFC training in a conditioning chamber (context A) without Dox to label c-*fos*-positive ensembles with ChR2. After 24 h, activation of ChR2-expressing c-*fos* ensembles using blue light illumination under a distinct neutral context (context B) elicited freezing responses only during illumination, suggesting that reactivation of c-*fos* ensembles formed during CFC training was sufficient for the retrieval of the fear memory (**Figure 1B**). Inactivation of c-*fos*-positive ensembles in the dorsal CA1 region of the hippocampus expressing Arch-T has also been shown to impair memory recall (Tanaka et al., 2014). Similar to c-*fos* studies, optogenetic suppression of neuronal activity of *Arc*-positive neurons in hippocampal CA3 or dentate gyrus (DG), labeled with Arch-T during CFC training (context A), significantly impaired memory retrieval during re-exposure to the identical context (**Figure 1C**; Denny et al., 2014). Conversely, mice showed intact memory retrieval during suppression of *Arc*-positive ensembles responding to context B, supporting the concept of specificity in *Arc*-expressing ensembles (Denny et al., 2014). These studies indicate that reactivation of IEG ensembles represents a critical event underlying retrieval of fear memories.

Optogenetic or pharmacogenetic activation of c-*fos*-positive neurons related to the neutral context B during fear memory training in context A interfere with memory encoding by generating a hybrid contextual fear memory (Garner et al., 2012; Ramirez et al., 2013). Such a hybrid contextual fear memory creates a “false” memory for a neutral context (Ramirez et al., 2013). These studies demonstrated the physiological importance of IEG-expressing neuronal ensembles in memory encoding and suggested that IEG expression during CFC training may help integrate the activated ensembles into the neuronal circuits for fear memory formation. It should be noted that, whereas the pharmacogenetic study employed a systemic agonist injection to activate c-*fos*-positive ensembles throughout the whole brain (Garner et al., 2012), the optogenetic studies used focal optical fiber implantation to manipulate activities of IEG-positive neurons within the relevant subregion of hippocampus (i.e., DG, CA3, or CA1; Liu et al., 2012; Ramirez et al., 2013; Denny et al., 2014; **Figure 2**). Although these pharmacogenetic and optogenetic manipulations yielded overall consistent behavioral outputs of fear memory recall, it remains elusive whether brain-wide pharmacogenetic activation and hippocampal subregion-specific optogenetic manipulation deal with the same content of memory as natural memory retrieval. Especially because each sub-hippocampal region has been shown to have a distinct function in processing spatial information (e.g., pattern separation in DG and pattern completion in CA3; Nakazawa et al., 2002; McHugh et al., 2007), artificial activity manipulation of IEG-expressing ensembles in specific hippocampal subregions may result in different types of memory and information processing.

Several studies attempted to associate memory traces in the hippocampus and basolateral amygdala (BLA) by optogenetic neuronal activation. Simultaneous optogenetic co-activation of c-*fos* ensembles in the BLA (responding to a fear experience) with c-*fos*-expressing CA1 neurons (encoding a neutral context) generated a new fear memory associated with the neutral context (Ohkawa et al., 2015). Similarly, c-*fos* ensembles in the DG encoding a rewarding context can be changed to a fear memory trace via optical reactivation of the DG reward-related ensemble during fear conditioning (Redondo et al., 2014). These data suggest that IEG-expressing ensembles in brain regions such as the hippocampus and amygdala integrate to create associative fear memory. Consistent with this theory, a recent study demonstrated a critical role of the hippocampal-neocortical network formed by IEG-expressing neurons (Cowansage et al., 2014). The authors first showed that pharmacological blockade of hippocampal activity impaired fear memory retrieval, consistent with previous observations (Kitamura et al., 2009; Wiltgen et al., 2010). When c-*fos*-positive ensembles in the retrosplenial cortex (RSC) formed during training were optogenetically reactivated, the impairment of fear memory retrieval caused by hippocampal activity blockade was restored (Cowansage et al., 2014), suggesting that hippocampal neurons may contribute to fear memory retrieval by reactivating c-*fos*-positive memory ensembles distributed in the neocortical areas, including the RSC. Furthermore, suppression of hippocampal c-*fos* ensemble reactivation resulted in failure of the reactivation of c-*fos* memory ensembles in several cortical regions (Tanaka et al., 2014), indicating that IEG-expressing neurons distributed across distinct brain regions directly or indirectly activate each other and are crucial to forming and/or recalling a fear memory (Cowansage et al., 2014; Tanaka et al., 2014; Matsuo, 2015).

These hippocampal-cortical memory trace interactions at the early phase of memory formation and maintenance may stimulate reconsideration of a conventional view of system consolidation that assumes a slow, sequential involvement of cortical areas in memory trace formation (Frankland and Bontempi, 2005). Indeed, recent studies have shown hippocampal-dependent learning tasks immediately induce IEG expression in the cortical areas (Tse et al., 2011; Czajkowski et al., 2014). Furthermore, cortical activation in an early phase of memory formation induces functional changes in cortical neurons (Bero et al., 2014) and contributes to late-phase (remote) memory formation (Lesburgueres et al., 2011), suggesting that early phase (recent) memory trace exists in the neocortex as well as the hippocampus. The observations of Cowansage et al. (2014), in which the activation of c-*fos* ensembles in the RSC could evoke fear responses even in the absence of hippocampal activity, support the parallel encoding processing of fear memory in the neocortex and in the hippocampus shortly after learning. These results do not indicate, however, that hippocampal memory trace is unnecessary for natural retrieval processes, because “artificial” cortical activation could be sufficient to override the requirement of the hippocampus for contextual memory.

### c-*fos* Memory Trace Largely Overlaps with Other IEG-Expressing Ensembles

The majority of studies concerning memory trace have focused on cell ensembles expressing c-*fos* (**Figure 2**). Do c-*fos*-expressing ensembles also express other IEGs? Surprisingly, little information is available regarding how and to what extent c-*fos*-expressing neuronal ensembles overlap with neuronal ensembles expressing other IEGs during memory encoding, although the individual expression patterns of each IEG have been well documented. Double *in situ* hybridization (ISH) analysis on the same section indicated most neurons in the cortical regions coexpressed IEGs including c-*fos*, *Arc*, and *Nr4a1* after monocular stimulation or sleep deprivation (Thompson et al., 2010; Nakagami et al., 2013). In the hippocampal CA1 and CA3, *Arc*-expressing neurons responding to a context exposure were more largely overlapped with *Homer1a*-expressing ensembles responding to the same context exposure than to a different context exposure (Vazdarjanova and Guzowski, 2004). Immunofluorescence analysis of fear-conditioned brain sections revealed that most DG neurons coexpressed *Arc* and Egr-1 regardless of their differential expression time courses (Lonergan et al., 2010). These studies suggest that most neuronal ensembles encoding the fear memory may simultaneously express various IEGs, although the extent to which IEG-positive ensembles responding to behavioral tasks overlap with other IEGs-positive ensembles remains elusive.

### Functional Characteristics of IEG-Expressing Neurons

Recent studies have investigated the characteristics of IEG-expressing neurons when compared to non-expressing neurons. In cortical regions, increased spontaneous firing rates have been observed in somatosensory neurons expressing c-*fos* or *Arc* (Yassin et al., 2010), and *Arc*-expressing neurons in the frontal cortex exhibit persistent firing after motor learning (Ren et al., 2014). Fear conditioning has been shown to increase surface expression of calcium-permeable AMPARs (i.e., GluA1 subunit-containing AMPA receptors) within c-*fos*-expressing cortical neurons (Descalzi et al., 2012). In the hippocampus, novel environment exposure alters dendritic spine morphology in *Arc*-expressing hippocampal neurons (Kitanishi et al., 2009), while fear conditioning induces selective spine elimination in CA1 neurons expressing c-*fos* (Sanders et al., 2012). In contrast to CA1 neurons, DG neurons expressing c-*fos* exhibit showed the increased spine density and enhanced synaptic transmission associated with protein synthesis after CFC training (Ryan et al., 2015). Although these studies demonstrated that experience-dependent IEG induction correlated with functional changes in activated neurons, it remains unclear whether IEG expression induces the functional changes required to generate memory traces.

## The Roles of *Arc* in Synaptic Plasticity and Memory Formation

Although optogenetic and pharmacogenetic interventional approaches have suggested an important involvement of IEG-expressing neurons in memory formation, it remains unclear how IEG expression during learning participates in incorporation of IEG-expressing ensembles into the memory trace. In contrast to studies of memory traces encoded by c-*fos*-positive ensembles, little is known concerning the biological and physiological effects of c-*fos* on synaptic plasticity and neuronal circuit reorganization, in part because c-*fos* encodes a transcription factor composing the AP-1 complex, whose target genes in neurons have yet to be fully characterized. In contrast to c-*fos*, several IEGs including *BDNF*, *narp*, *homer*1a, and *Arc* are known to encode synaptic or secretory proteins directly affecting synaptic properties (Chowdhury et al., 2006; Chang et al., 2010; Roloff et al., 2010; Lu et al., 2013). Elucidation of these proteins would shed light on molecular mechanisms underlying the incorporation of IEG-expressing ensembles into the memory trace. In the following sections, we review recent findings regarding the role of *Arc* in synaptic plasticity and memory formation.

### Long-Term Memory Formation Requires *Arc* Induction

*Arc* expression is required for LTM consolidation, but not for learning or short-term memory formation (STM; Plath et al., 2006). *Arc* knockout (KO) mice exhibit impaired consolidation of spatial and fear memories (Plath et al., 2006; Peebles et al., 2010; Yamada et al., 2011). Transient inhibition of *Arc* expression following infusion of *Arc* antisense oligodeoxynucleotides (ODNs) into the hippocampus, lateral amygdala, or anterior cingulate cortex inhibits memory consolidation (Guzowski et al., 2000; Ploski et al., 2008; Holloway and McIntyre, 2011; Nakayama et al., 2015). This memory impairment occurs only when *Arc* antisense ODNs are infused immediately following memory acquisition, suggesting that induction of *Arc* expression in response to training experience is necessary for LTM formation.

### Synaptic Localization of *Arc*

One unique characteristic of *Arc* is that its mRNA and protein can be targeted to dendritic compartments of neurons. Within minutes of neuronal activation triggered by behavioral events, *Arc* mRNA is expressed in the nucleus and subsequently transported through the cytoplasm into the dendrites (Wallace et al., 1998; Guzowski et al., 1999). Localization of dendritic mRNA is also regulated by synaptic activity; both *Arc* mRNA and protein accumulate in activated dendrites receiving high frequency stimulus (HFS; Steward et al., 1998; Moga et al., 2004). Biochemical and electron microscopic studies have confirmed the post-synaptic localization of *Arc* protein (Moga et al., 2004; Rodriguez et al., 2005; Chowdhury et al., 2006). Since *Arc* protein does not have obvious catalytic or other known functional domains, it is believed to function by interacting with other post-synaptic proteins. *Arc* interacts with endophilin and dynamin to form an endocytic complex believed to be involved in post-synaptic AMPAR trafficking, since *Arc* overexpression or knockout decreases or increases surface AMPAR expression, respectively (Chowdhury et al., 2006). Moreover, the effect of *Arc* on AMPAR regulation disappears when the interaction of *Arc* with endophilin/dynamin is disrupted (Chowdhury et al., 2006; Rial Verde et al., 2006). Although *Arc* facilitates AMPAR endocytosis, it does not appear to directly interact with AMPAR. A recent study revealed that the interaction of *Arc* with TARPγ2 (stargazin) is required for *Arc*-dependent AMPAR synaptic scaling (see below; Zhang et al., 2015).

### Roles of *Arc* in Synaptic Scaling and Synaptic Plasticity

Neurons possess the ability to maintain their excitability within a certain dynamic range by modifying surface AMPAR expression on synapses in response to changes in synaptic input, without affecting the relative balance between strong and weak synapses. These cellular changes have been termed “homeostatic” plasticity (Turrigiano, 2008), and induction of *Arc* by neuronal activation and synaptic AMPAR endocytosis provide this IEG with a function in this process. *Arc* KO neurons in culture lack homeostatic AMPAR scaling (Shepherd et al., 2006) and *Arc* KO mice exhibit a deficit of synaptic scaling in response to sensory deprivation (McCurry et al., 2010). The expression of *Arc* appears to be required for cellular synaptic scaling in response to neuronal activity.

**FIGURE 3:**
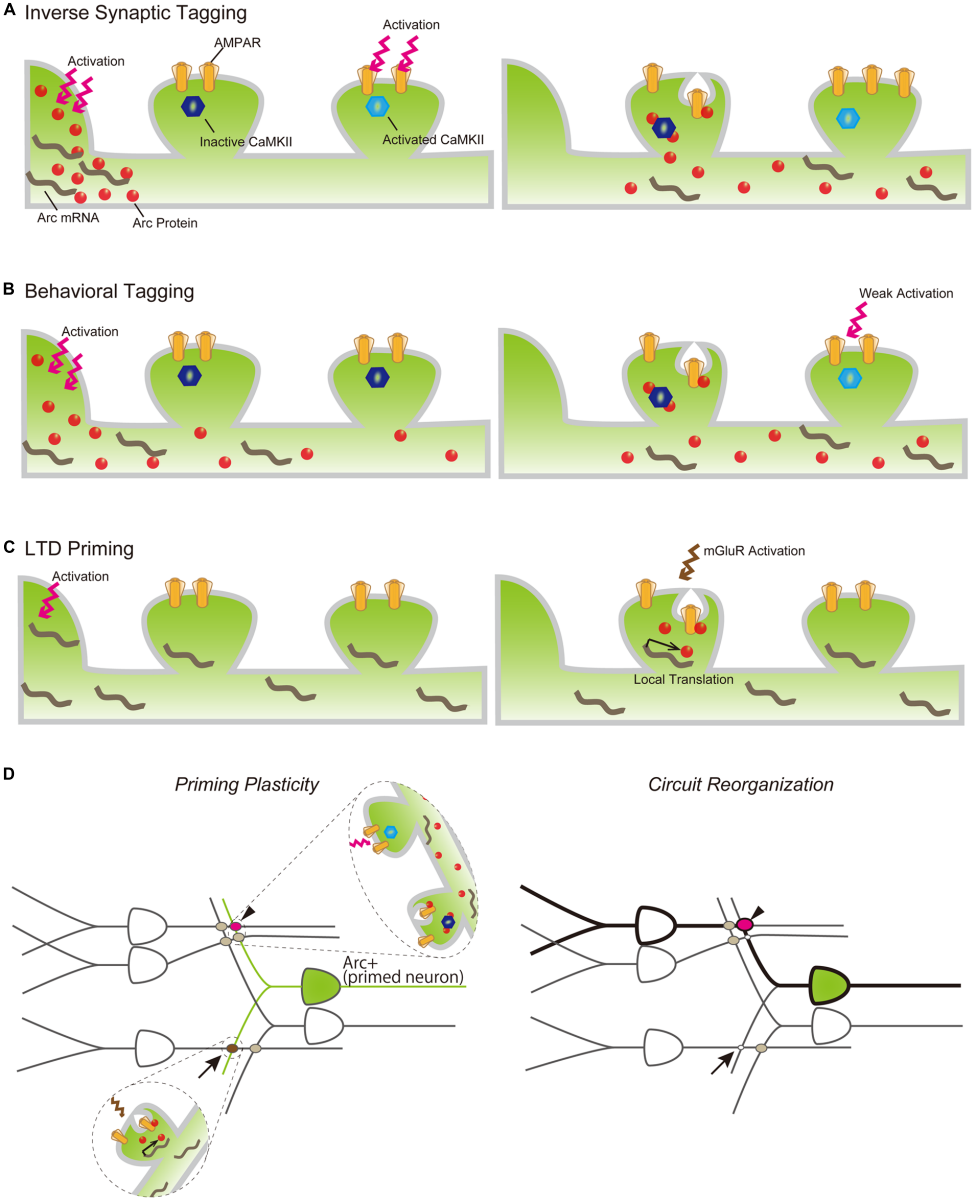
**Models of *Arc* mRNA and protein dynamics in inverse tagging **(A)**, behavioral tagging **(B)**, and LTD priming **(C)** processes.** **(A)** *Arc* protein is synthesized in the soma following neuronal activation (left) and transported to an activated dendrite (right). *Arc* preferentially binds to the inactive form of CaMKII and promotes AMPAR endocytosis in the inactive synapse, resulting in the increasing synaptic strength of the activated synapse. **(B)** A novel exploration task induces *Arc* protein expression (left). *Arc* protein diffuses throughout the dendrite, decreases AMPAR in the non-tagged synapse, and serves to maintain the enhanced synaptic strength of synapses transiently potentiated by a weak training (right). **(C)** Other types of behavioral experiences promote *Arc* mRNA synthesis in the nucleus and transport to nearby synaptic sites, but its translation is suspended (left). Subsequent mGluR activation promotes *Arc* translation from dendritically localized *Arc* mRNA, resulting in AMPAR internalization and LTD of the mGluR-activated synapse. **(D)** *Arc* expression plays a permissive role in inducing synapse-specific plastic changes to organize a new memory trace following a new behavior/experience. Arrow and arrowhead indicate LTD-primed and behavioral-tagged synapses, respectively.

*Arc*-dependent AMPAR endocytosis is also involved in induction of LTD (Plath et al., 2006; Park et al., 2008). Activation of metabotropic glutamate receptors (mGluRs) rapidly induces *Arc* translation, which is necessary for expression of mGluR-dependent LTD (Park et al., 2008) and suggests that *Arc* protein plays an important role in both input-specific synaptic plasticity and cell-wide synaptic scaling. Synaptic AMPAR downregulation by *Arc* appears irreconcilable with increases in *Arc* expression reported to occur following LTP-inducing stimulus and the transport of *Arc* mRNA and protein into activated dendrites. However, recent observations that *Arc* protein is preferentially transported to inactive dendritic spines by binding with the inactive form of CaMKIIβ and that AMPAR is selectively decreased in inactive spines in which *Arc* is accumulated (Okuno et al., 2012), may help explain this apparent incongruity. This *Arc*-dependent downregulation of AMPAR in inactive synapses, termed “inverse synaptic tagging,” likely functions to increase the contrast of synaptic strength between active and inactive synapses following synaptic potentiation (**Figure 3A**). Taken together, consolidation of synaptic plastic changes responding to neuronal activity is achieved, in part, via regulation of the expression and localization of *Arc* protein, which is in turn involved in surface AMPAR endocytosis.

### Metaplastic Changes of *Arc*-Expressing Neurons

While it has been well documented that *Arc* expression is robustly induced throughout the rodent brain following exposure to a novel environment, the significance and consequence of its upregulation remain unclear. Several studies have revealed that *Arc* induction is associated with alteration of neuronal network properties, thereby facilitating consolidation of otherwise labile memories. For example, *Arc*-expressing neurons responding to novel environment exposure are preferentially reactivated during subsequent spontaneous hippocampal ripples, which are crucial for memory consolidation (Mizunuma et al., 2014). *Arc* may also be involved in a specific type of memory facilitating process called “behavioral tagging” (Moncada and Viola, 2007; Ballarini et al., 2009; Wang et al., 2010; Moncada et al., 2011). During “behavioral tagging,” weak training tasks inducing STMs that last for only a few hours can produce LTM that lasts for over 24 h when accompanied by a novel experience that stimulates protein synthesis. This experience-dependent LTM facilitation is assumed to rely on PRPs synthesized during the novel experience, which would serve to induce and maintain plastic changes in the synapses “tagged” during the weak training. The suppression of *Arc* induction using ODNs during a novel experience inhibited LTM formation of weak behavioral tasks following the novel experience (Martinez et al., 2012), indicating that *Arc* may function as a PRP to facilitate LTM formation. Together with the inverse tagging mechanism described above, *Arc* protein synthesized prior to weak training may enhance synaptic strength between potentiated and non-potentiated synapses during the training (**Figure 3B**). These findings suggest that *Arc* expression prior to a behavioral task may influence the priming of synaptic plasticity. The history of neuronal activation can facilitate LTP or LTD induction, e.g., antecedent neuronal activities can alter the threshold of LTP and/or LTD expression induced by subsequent inputs. This type of plastic change is called “metaplasticity” (Abraham, 2008), and *Arc* has been suggested to be a modulating factor in this process (Shepherd and Bear, 2011). To this end, *Arc* induction has been reported to facilitate LTD expression (Jakkamsetti et al., 2013). *Arc*-expressing hippocampal neurons responding to exposure to a novel environment showed unaltered excitatory synaptic responses compared with the surrounding non-expressing neurons, but also showed prime mGluR-dependent LTD. Exposure to a novel environment also promoted expression and transportation of *Arc* mRNA to activated dendrites, while subsequent mGluR stimulation induced the translation of *Arc* in stimulated synapses and facilitated AMPAR endocytosis to express LTD in primed neurons (Jakkamsetti et al., 2013; **Figure 3C**). Furthermore, repeated experience caused a decrease in synaptic inputs to activated neurons via the *Arc* LTD-priming mechanism (Jakkamsetti et al., 2013). Taken together, LTD priming by *Arc* contributes to synaptic reorganization, which may help establish spatial recognition. Behavioral tagging and LTD priming by *Arc* are not mutually exclusive and can occur in the same neurons simultaneously. *Arc* induction may also increase the flexibility of synaptic changes in responding to various forms of subsequent stimuli. For example, synapses activated during repeated exposure to the same environment will be weakened by priming and induction of LTD (Jakkamsetti et al., 2013; **Figure 3D**, arrow). Conversely, synaptic tagging and inverse tagging processes will heighten the contrast of synaptic strength in response to weak synaptic inputs (**Figure 3D**, arrow head). These synaptic modifications of *Arc*-expressing neurons will reorganize the activated circuits underlying memory trace formation (**Figure 3D**, right).

## Conclusion and Future Directions

In this review, we characterized the role of IEG-expressing neurons in memory formation and storage. Neurons activated during cognitive tasks induce IEGs and organize the memory trace (**Figures 1** and **2**). During memory formation and recall, hippocampal IEG-positive cells are co-activated alongside IEG-positive neurons in other brain areas, including the amygdala and neocortex, suggesting that IEG-positive neurons are preferentially connected with each other across brain regions. Furthermore, IEG-positive neurons exhibit functional synaptic changes that may underlie memory formation. We also summarized evidence for the role of *Arc* in synaptic plasticity and memory formation. Taken together, the literature suggests that continued characterization of the functional changes in *Arc*-expressing neurons will elucidate novel molecular mechanisms underlying memory formation and/or storage.

The expression of IEGs is dynamically regulated in response to neuronal activity in the brain; while many neurons only express IEG at basal levels, some neurons display rapid induction of IEG expression surpassing the basal level after learning (Rosen et al., 1998; Guzowski et al., 1999; Vann et al., 2000; Hall et al., 2001; Ramirez-Amaya et al., 2005). Such dual components of IEG expression raise an important but unsolved question: what is the significance of learning-induced, but not basal, IEG expression? Our current knowledge about IEG functions in memory largely depends on loss-of-function studies, most of which use conventional IEG KO animals. Such approach could demonstrate the necessity of IEGs, but not specifically learning-induced IEGs, in learning and memory. To address this issue, one possible strategy is to specifically suppress learning-induced IEGs by directly manipulating activity-dependent expression. The promoters and enhancers that contribute to activity-dependent expression of c-*fos*, *Arc*, and *Bdnf* have been characterized (Robertson et al., 1995; West et al., 2001; Kawashima et al., 2009). In fact, such a strategy has successfully dissected the role of activity-dependent expression of the *Bdnf* gene (Hong et al., 2008; Sakata et al., 2009, 2013). *Bdnf* possesses multiple promoters including promoter IV, which is responsible for activity-dependent *Bdnf* expression. When promoter IV is mutated, activity-dependent, but not basal, *Bdnf* expression is greatly reduced and cortical circuit organization is altered (Hong et al., 2008; Sakata et al., 2009). Furthermore, hippocampal synaptic plasticity and behavioral flexibility are impaired (Sakata et al., 2013). Manipulations in the transcriptional *cis*-elements would therefore help elucidate a causal relationship between behaviorally related IEG induction and memory trace formation.

Recently, visualization of dynamic changes in IEG expression *in vivo* using fluorescent reporters under the control of the IEG promoter has been achieved (Barth et al., 2004; Wang et al., 2006; Eguchi and Yamaguchi, 2009; Grinevich et al., 2009; Kawashima et al., 2009). These techniques enable us to analyze neurons expressing IEGs while responding to behavioral tasks, facilitating the understanding of the physiological roles of activity-dependent IEG expression in synaptic plasticity and memory formation.

Furthermore, it is widely believed that dysregulation of neuronal activity and synaptic functions cause various types of cognitive disorders including autism, schizophrenia, and dementia, as these diseases are related to mutations in genes associated with activity-dependent gene expression and synaptic maturation (West and Greenberg, 2011; Purcell et al., 2014). Future studies analyzing the roles of activity- and/or behavioral-dependent expression of *Arc* and other IEGs in synaptic plasticity may enhance our understanding of the pathogenesis and treatment of specific psychiatric and neurological disorders.

## Author Contributions

KM, conceived the content and wrote the manuscript; MA, provided the ideas and discussions; HO, conceived the content, wrote the manuscript, and supervised the work.

## Conflict of Interest Statement

The authors declare that the research was conducted in the absence of any commercial or financial relationships that could be construed as a potential conflict of interest.
